# Application study of curcumin fluorescent complex coated with pharmaceutical excipients for cell imaging

**DOI:** 10.3389/fchem.2023.1153729

**Published:** 2023-03-16

**Authors:** Chen Shao, Xiaoli Zhang, Shihe Shao, Feng Jin

**Affiliations:** ^1^ Affiliated Hospital of Jiangsu University, Zhenjiang, China; ^2^ Department of Critical Care Medicine, Affiliated Yixing People’s Hospital, Jiangsu University, Yixing, China; ^3^ Medical College of Anhui University of Science and Technology, Huainan, China

**Keywords:** curcumin, β-cyclodextrin, acrylic resin, pharmaceutical excipients, cell imaging

## Abstract

Taking curcumin as the starting point, β-cyclodextrin was introduced on both sides, and lipid-soluble curcumin was coated by acrylic resin using oil-in-water strategy. Four different types of curcumin fluorescent complexes EPO-Curcumin (EPO-Cur), L100-55-Curcumin (L100-55-Cur), EPO -Curcumin-β-cyclodextrin (EPO-Cur-β-cd) and L100-55-Curcumin-β-cyclodextrin (L100-55-Cur-β-cd) were prepared to solve their own solubility and biocompatibility issues. The prepared curcumin fluorescent complexes were characterized and tested by spectroscopy. The characteristic peaks of 3446 cm^−1^ (hydroxyl group), 1735cm^−1^(carbonyl group) and 1455 cm^−1^ (aromatic group) were determined in the infrared spectrum. In the fluorescence emission spectrum, it was found that the emission intensity of different curcumin fluorescent complexes in polar solvents reached hundreds of times. Through the transmission electron microscopy shows that acrylic resin tightly coats curcumin into rods or clusters. In order to observe their compatibility with tumor cells more directly, live cell fluorescence imaging was carried out, and it was found that all four kinds of curcumin fluorescence complexes had good biocompatibility. In particular, the effect of EPO-Cur-β-cd and L100-55-Cur-β-cd is better than that of EPO-Cur and L100-55-Cur.

## 1 Introduction

The important characteristic and active components extracted from the traditional chinese medicine turmeric have good application prospects and clinical therapeutic potential in the diversity of pharmacological actions (Kocaadam and Sanlier 2017; Hosseini and Hosseinzadeh 2018; Hay et al., 2019; Liu et al., 2022a). In a number of studies involving cancer and tumor cells, it was found that curcumin may play a good inhibitory role in a variety of different links (Mortezaee et al., 2019; Zendehdel et al., 2019), it effectively inhibits the uncontrolled metastasis of cancer cells (Jia et al., 2019), the proliferation of tumor cells, the activity of cancer-inducing chemicals in the stomach and colon (Porras et al., 2020; Naji et al., 2021), and inhibits angiogenesis by modulating protease activity (Binion et al., 2008; Liu et al., 2022b; Hu et al., 2022). However, curcumin has some disadvantages such as poor water solubility, low bioavailability and poor gastrointestinal absorption (Sabet et al., 2021; Sohn et al., 2021). In order to solve this problem, new drug delivery systems such as liposomes, micelles and structural modifications were developed (Lian et al., 2020). For example, curcumin liposomes mixed with drugs improved the bioavailability (Hong et al., 2020), and the structure of curcumin was modified to synthesize new curcumin analogues based on the natural product curcumin (Xu et al., 2018; Noureddin et al., 2019; Zhao et al., 2019; Purushothaman et al., 2022). Although these methods improved the solubility of curcumin to a great extent, they did not achieve the expected effect due to the complicated preparation process and high cost. In view of the pleiotropic structure of curcumin itself, the delivery route of the composite drug is prepared by modifying the main structure and the coating of pharmaceutical excipients.

The research and application of acrylic resin-based pharmaceutical excipients are very extensive, especially in the field of pharmacy (Bettencourt et al., 2010; Villanova et al., 2011; Liu S. et al., 2022). In view of the shortcomings of many drugs directly administered, coating technology can effectively solve such problems. The use of coating technology can increase the solubility of drugs with low solubility, which can be better absorbed and improve the relative bioavailability in the human body (Kausar 2018; Haining et al., 2022). Not only that, but coating technology can also reduce the damaging effects of drugs on the body during treatment. Therefore, coating technology is also often applied to various formulations such as tablets, liquid formulations and the like (Katona et al., 2016; Desai et al., 2018; Boase et al., 2022; Jing et al., 2022; Shao et al., 2022; Xiao et al., 2022).

In order to solve the above points, and based on the known active drugs, this project designed a novel curcumin fluorescent polymer complex with polypropylene material as the core carrier. 1) The hydroxyl functional groups on both sides of curcumin were modified to introduce β-cd to increase its solubility; 2) Once again, acrylic resin was used to coat curcumin β-cd complex to further solve the problem of biocompatibility and absorption rate, and retain and inherit the efficacy and role of its main drug. To realize the development of simple, efficient and practical curcumin fluorescence complex.

## 2 Experiment

### 2.1 Materials and instruments

All solvents and reagents were purchased commercially and used without further purification. The reagents used are curcumin (98%, RG) and beta-cyclodextrin (98%, RG), which were purchased through commercial channels such as Titan Technologies. Full-wavelength absorption spectra were recorded using a UV-2550 spectrophotometer. Under excitation at 490 nm, the fluorescence emission spectrum of Shimadzu RF-5301pcs spectrophotometer was measured. All optical measurements were performed at room temperature.

### 2.2 Synthesis

**SCHEME 1:**
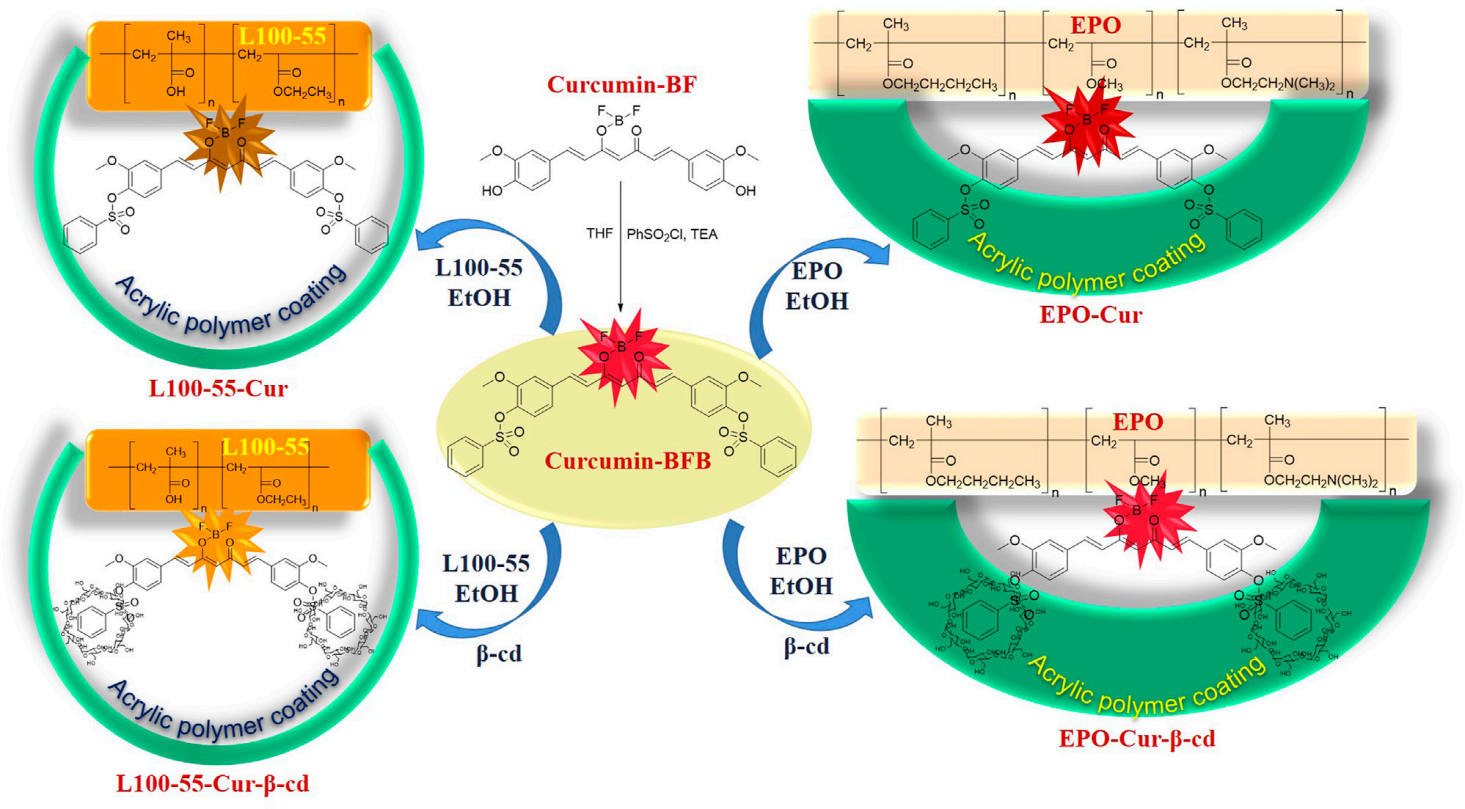
Synthetic route of L100-55-Cur, L100-55-Cur-β-cd, EPO-Cur and EPO-Cur-β-cd.

Curcumin-BF (1.0 g, 2.7 mmol) and of benzenesulfonyl chloride (0.8 ml, 6.3 mmol) were dissolved in tetrahydrofuran (THF) (8 ml), triethylamine (0.9 ml, 6.8 mmol) was added, and then at the room temperature stirred for 30 min. The reaction was confirmed by thin layer chromatography. The THF was rotary evaporated and ultrapure water was added to precipitate the solid. After stirring the solid with methanol for 30 min, 1.7 g of curcumin-BFB was obtained (Scheme 1).

Take a 50 ml round-bottom flask 0.1 g of curcumin-BFB and 0.1 g of L100-55 were dissolve in EtOH (5 ml), and then at the room temperature stirred for 6 h s. The EtOH was concentrated to afford 0.88 g of **L100-55-Cur** (Scheme 1).

**L100-55-Cur-β-cd**, **EPO-Cur** and **EPO-Cur-β- cd** were obtained by the same method.

### 2.3 FT-IR

Grind potassium bromide into powder in an agate mortar under infrared light, then press potassium bromide into transparent uniform flakes using a tablet press. Place the potassium bromide sheet on the sample holder of the spectrometer, start the measurement, and obtain the background spectrum. The samples were then pulverized together with potassium bromide, formed into transparent uniform flakes with a tablet machine, and analyzed by infrared spectroscopy.

### 2.4 Spectroscopic properties

#### 2.4.1 UV spectrum

Ultraviolet–visible (UV–vis) spectra were recorded on a UV-2550 spectrophotometer using a 1 cm path length quartz cuvette and fluorescence Spectra were performed on Shimadzu RF-5301PCS Spectro fluorophotometer at room temperature. A proper amount of the compound was dissolved in THF and prepared into 1 mM mother liquor for later use. Spectral tests of solutions with different concentrations were prepared according to needs and data were recorded. The UV–Vis wavelength range is 400–700 nm. The fluorescence of the compounds were obtained at the optical path of 10 mm and the excitation wavelength of 450 nm, and the wavelength range of the recorded emission was 400–800 nm.

#### 2.4.2 Fluorescence emission spectrum

Dissolve the four polymers in dichloromethane (DCM), make them into a series of concentrations, and then conduct fluorescence spectrum test in dimethylsulfoxide (DMSO), ethylacetate (EA), methanol (MeOH) and tetrahydrofuran (THF) solution environment. Set the excitation wavelength as the corresponding maximum absorption wavelength in ultraviolet. The corresponding fluorescence emission spectrum was obtained.

#### 2.4.3 Cellular uptake and localization by transmission electron microscope

Transmission Electron Microscope (TEM) was performed on a Zeiss Ultra Plus at an accelerating voltage of 15 keV, with an attached Oxford Instruments X-Max 60 mm2 SDD X-ray microanalysis system. The ethanol suspended precipitate of the sample was added to a silicon wafer, and the sample was attached to a sample tray with a conductive adhesive, and TEM images were obtained using a scanning electron microscope at 2.0 μm and 200 nm rulers, respectively. A thin supporting film is adhered on the copper net in advance, and a proper amount of powder and tetrahydrofuran are added to the small beaker respectively, and ultrasonic oscillation is carried out for 10–30 min. After 3–5 min, the uniform mixed liquid of powder and tetrahydrofuran is sucked by a glass capillary tube, and then two to three drops of the mixed liquid are dropped onto the copper net and dried. Wait for more than 15 min to volatilize tetrahydrofuran as much as possible. Finally, put the sample on the sample table and insert it into the electron microscope for observation.

#### 2.4.4 Cell imaging

HeLa cells in logarithmic growth phase were treated with trypsin, seeded in a 96-well plate with a circular cover, placed in a 5% CO_2_ incubator, and cultured at 37°C for 24 h to adhere. The prepared polymers EPO-Cur, EPO-Cur-β-cd, L100-55-Cur and L100-55-Cur-β-cd stock solutions (5 mg/ml) were prepared in DMSO, respectively, and then diluted with DMSO to prepare appropriate concentrations of solution. The cells in the original culture medium were removed in each of the different samples and replaced with a medium containing 5 μg/ml for 36 h. Afterwards it was discarded with PBS, washed twice, and then fixed with paraformaldehyde for 20 min after. The solution removed the repair solution with PBS and washed twice, incubated in DAPI dark room for 20 min, discarded the staining solution, washed 2 times with PBS, treated with anti-fluorescence inactivating scaffolds, and obtained fluorescent images of cells on a fluorescence microscope.

## 3 Results and discussion

**FIGURE 1:**
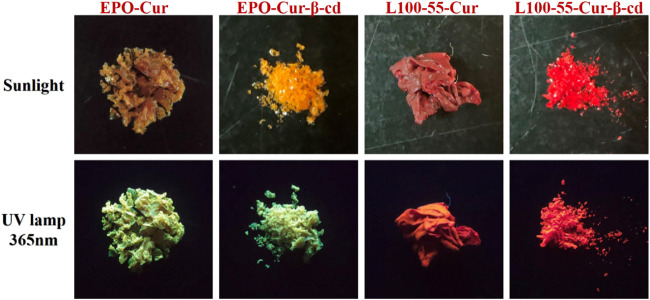
Sunlight and 365 nm images of four curcumin fluorescence complexes.

Starting from the design of oil-in-water concept and drawing on the existing research basis, benzenesulfonyl chloride is firstly introduced from both sides of the main skeleton structure of curcumin, and then β-cd is used to embed the benzenesulfonyl groups on both sides to increase its solubility in water and improve bioavailability Finally, the curcumin complex was coated with acrylic resin to play a key role in physical properties, and finally different series of target compounds L100-55-Cur, L100-55-Cur-β-cd, EPO-Cur and EPO-Cur-β-cd were prepared (Scheme 1 and Figure 1).

**FIGURE 2:**
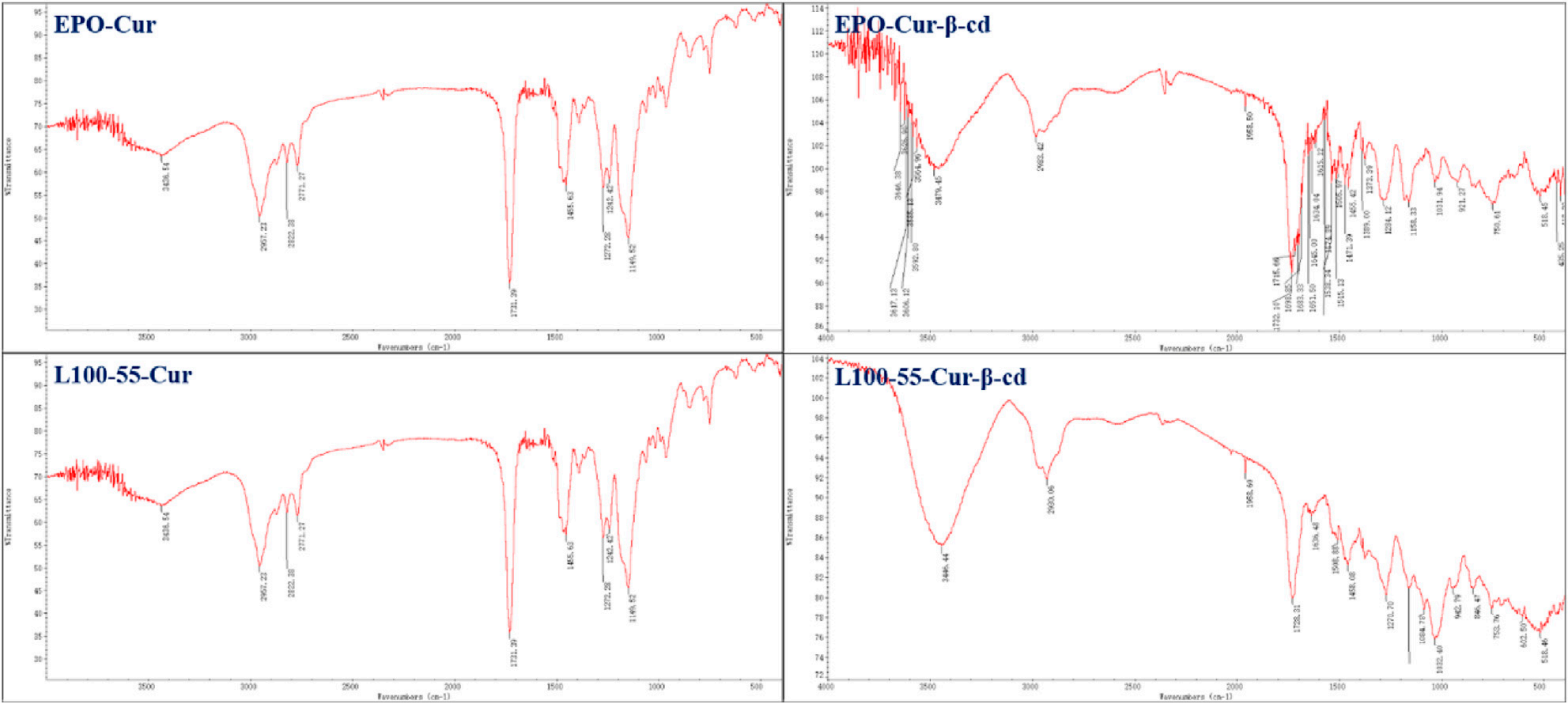
FT-IR absorption spectrum and cyclodextrin embedding characteristic peaks of four curcumin fluorescent complexes (Its scanning times are 16 and resolution is 4.0 cm^−1^).

In the infrared spectrum, a strong absorption peak can be observed in the range of 3446–3479 cm^−1^. This is because the two sides of the main structure of L100-55-Cur-β-cd and EPO-Cur-β-cd are embedded by β-cd and thus have characteristic peaks of hydroxyl groups, while L100-55-CUR and EPO-CUR do not have obvious absorption peaks compared with the previous two complexes. The strong absorption peak in the 1735 cm^−1^ range is the characteristic absorption peak of the two carbonyl groups of curcumin, while the subsequent absorption peaks in the 1635–1455 cm^−1^ range belong to the aromatic absorption peaks (Figure 2).

**FIGURE 3:**
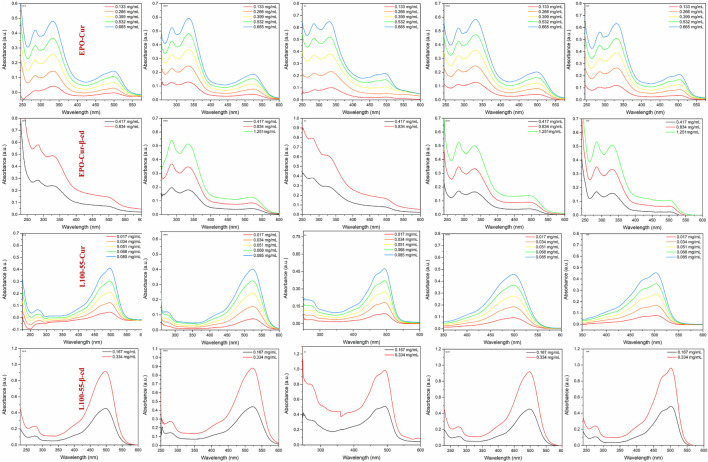
UV absorption spectrum of complexes L100-55-Cur, L100-55-Cur-β-cd, EPO-Cur and EPO-Cur-β-cd in different solvents.

In order to observe the spectral characteristics of the four complexes, dichloromethane, dimethyl sulfoxide, ethyl acetate, methanol and tetrahydrofuran were used as solvents for UV spectrum analysis. The results showed that the UV absorption spectra of EPO-Cur and EPO-Cur-β-cd complexes appeared multiple peaks, with the same maximum absorption peaks in the range of 495–512 nm. The UV absorption spectra of L100-55-Cur and L100-55-Cur-β-cd complexes are different from EPO-Cur and EPO-Cur-β-cd, which are single peaks, and the maximum absorption wavelength is in the range of 497–526 nm, the absorption intensity also increases with the concentration (Figure 3 and Table 1).

**TABLE 1:** Absorption and emission spectra data in different solutions of four complexes.

Complex	Items	Solvents				
		DCM	EA	THF	DMSO	MeOH
EPO-Cur	λ_abc_/nm	497	497	503	524	497
	λ_em_/nm	561	548	551	593	571
EPO-Cur-β-cd	λ_abc_/nm	489	490	502	518	494
	λ_em_/nm	559	550	552	578	577
L100-55-Cur	λ_abc_/nm	496	494	502	523	498
	λ_em_/nm	565	549	553	595	573
L100-55-Cur-β-cd	λ_abc_/nm	496	493	502	522	497
	λ_em_/nm	558	547	555	586	573

**FIGURE 4:**
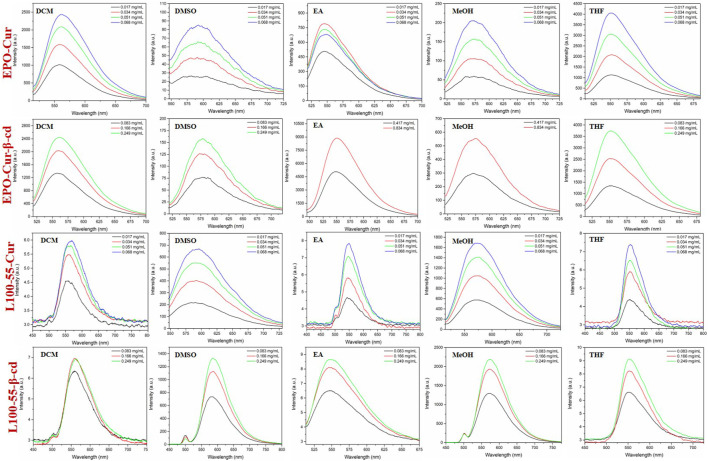
Fluorescence emission spectrum of complexes L100-55-Cur, L100-55-Cur-β-cd, EPO-Cur and EPO-Cur-β-cd in different solvents.

Likewise, the fluorescence emission spectra of these four complexes were determined in dichloromethane, dimethyl sulfoxide, ethyl acetate, methanol and tetrahydrofuran. The fluorescence emission spectra of EPO-Cur and EPO-Cur-β-cd complexes are in the range of 566–573 nm, and the emission intensity is basically between 2500 and 4000. The fluorescence emission spectra of L100-55-Cur and L100-55-Cur-β-cd complexes showed weak emission intensities in the range of 558–577 nm with emission intensities ranging from 6 to 2200. It is worth mentioning that the fluorescence emission intensity of EPO-Cur and EPO-Cur-β-cd is the weakest in polar solvents such as methanol and dimethyl sulfoxide, on the contrary L100-55-Cur and L100-55-Cur- β-cd is strongest in polar solvents. This is due to the difference in solubility due to the change in their physical properties due to their different coating materials, which results in a difference of several hundred times in emission intensity (Figure 4 and Table 1).

**FIGURE 5:**
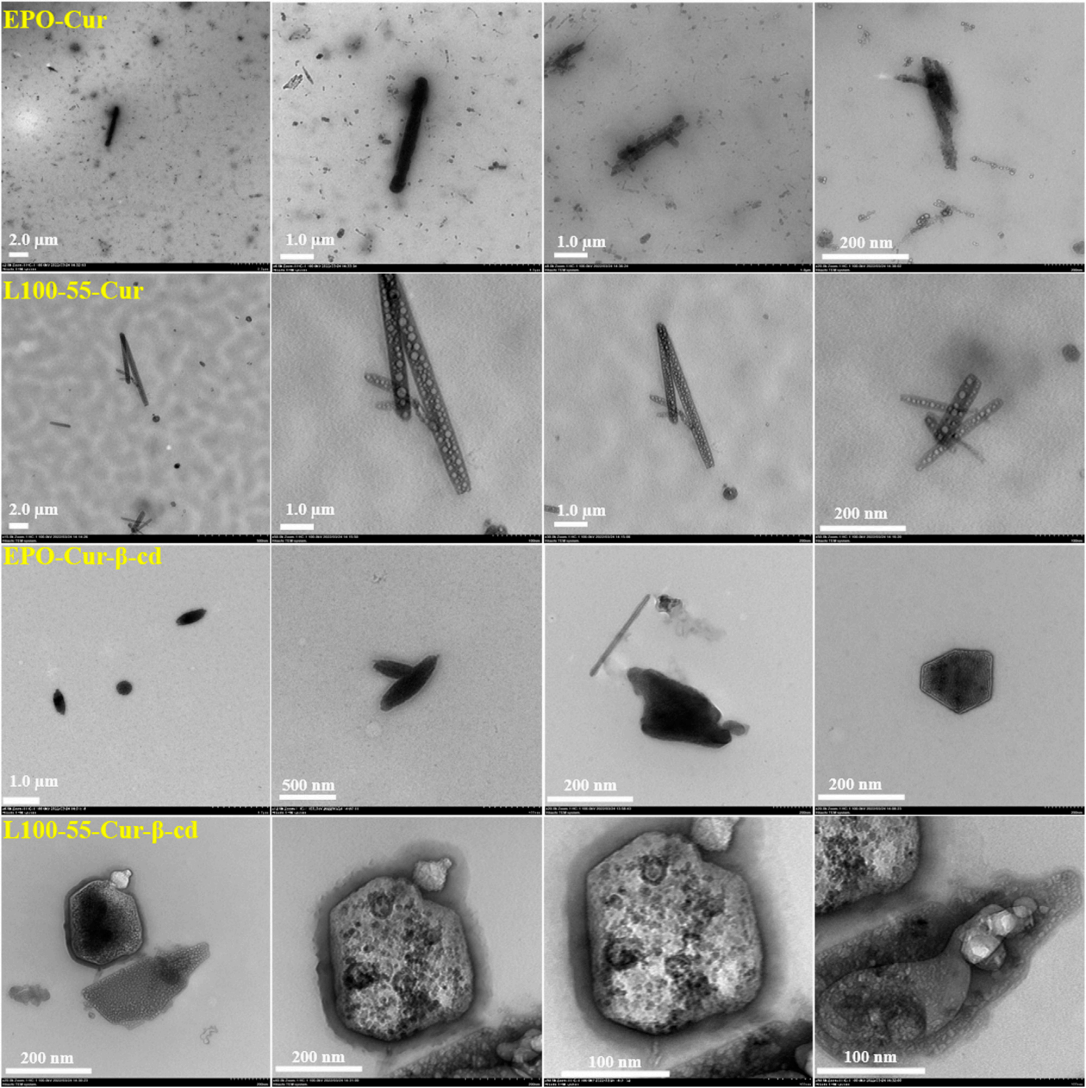
Transmission electron microscope images of complexes L100-55-Cur, L100-55-Cur-β-cd, EPO-Cur and EPO-Cur-β-cd.

To observe the internal morphology more directly, four composites were tested using transmission electron microscopy. It can be seen from the pictures that these complexes have strip- and cluster-shaped features depending on the coating material, and are clustered together. These four sets of images have very unique characteristics. For example, EPO-Cur and L100-55-Cur encapsulate numerous nanoparticles in strips, while EPO-Cur-β-cd and L100-55-Cur-β-cd are clearly clustered. This is because the former is acrylic resin -coated curcumin, while the latter is acrylic resin -coated curcumin and β-cd. These images show the physical properties of different types of complexes (Figure 5)**.**

**FIGURE 6:**
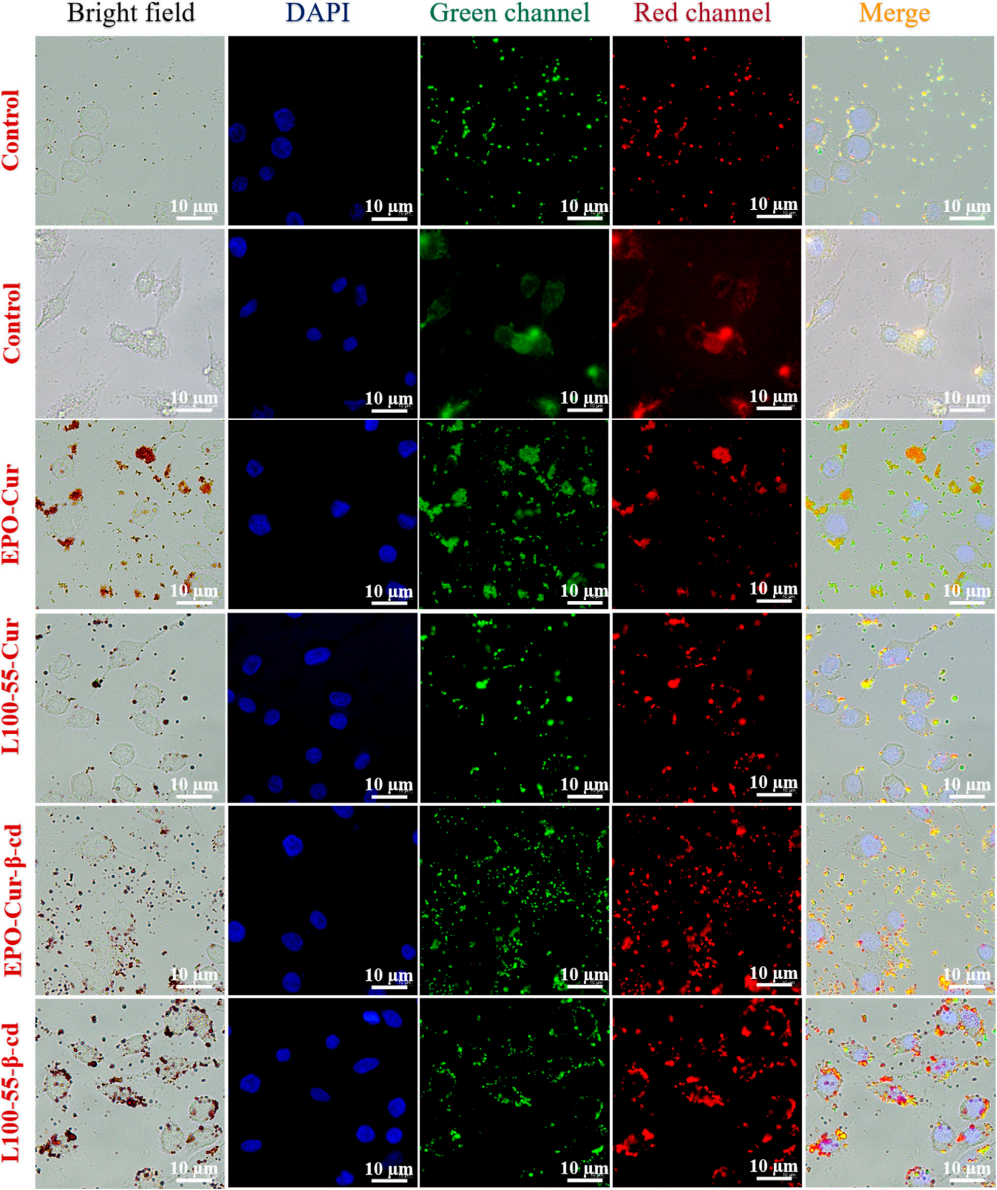
Cellular imaging of L100-55-Cur, L100-55-Cur-β-cd, EPO-Cur and EPO-Cur-β-cd.

The biocompatibility of the four complexes in tumor cells was investigated by cell imaging according to their acrylic resin coating characteristics. Staining of HeLa cells under confocal microscopy, the two control groups were clearly observed under fluorescence microscopy. As shown in Figure 6, bright field, DAPI, green channel, red channel, and merged imaging are provided. Compared with the two control groups, the biocompatibility of the four complexes with HeLa cells was significantly different. From the combined images, it can be found that the cell imaging effect of EPO-Cur-β-cd and L100-55-Cur-β-cd is better than that of EPO-Cur and L100-55-Cur Slightly obvious. This is because the coating of β-cd increases the solubility of the complex resulting in different effects between the two groups. Moreover, β-cd is often used as a pharmaceutical excipient to increase the stability of the drugs, improve the dissolution and bioavailability of the drugs, and reduce the toxic and side effects of the drugs (Figure 6). Therefore, by coating enteric-soluble polymer Eudragit@curcumin to improve its selectivity to cells and biocompatibility, to the water solubility and targeting of curcumin, and to the effect of cell imaging. Although the result is not very ideal, but to a large extent solved the problem of selectivity and compatibility with cells.

## 4 Conclusion

In summary, four kinds of curcumin fluorescence complexes were prepared using acrylic resin as drug excipients to improve the compatibility between drugs and cells. Although these complexes have improved the compatibility between curcumin and tumor cells to some extent, this is far from the current requirements. Compound preparation is an important research direction in recent years, especially the oil-in-water strategy will greatly increase the solubility and bioavailability of drugs. Therefore, curcumin has great bioavailability potential as an antitumor drug. Through the research of this subject, it is believed that with the in-depth discussion of curcumin preparations, it will provide a favorable reference for the subsequent research and development of new complex curcumin drugs.

## Data Availability

The original contributions presented in the study are included in the article/supplementary material, further inquiries can be directed to the corresponding authors.
